# Assessment of retinal and choroidal microcirculatory alterations following radiofrequency catheter ablation in atrial fibrillation patients using swept-source optical coherence tomography angiography

**DOI:** 10.3389/fcell.2025.1612303

**Published:** 2025-06-03

**Authors:** Jin Wang, Huiran Yang, Qingjian Li, Yucen Wang, Pei Zhang, Zhiliang Wang

**Affiliations:** ^1^ Department of Cardiology, Ninth People’s Hospital, Shanghai Jiao Tong University School of Medicine, Shanghai, China; ^2^ Department of Ophthalmology, Huashan Hospital, Fudan University, Shanghai, China; ^3^ Department of Ophthalmology, Shanghai Fourth Rehabilitation Hospital, Shanghai, China

**Keywords:** atrial fibrillation, optical coherence tomography angiography, vessel density, choroidal vascular index, choroidal thickness

## Abstract

**Purpose:**

This study employed swept-source optical coherence tomography angiography (SS-OCTA) to explore potential hemodynamic alterations in the retina and choroid following radiofrequency catheter ablation (RFCA) for atrial fibrillation (AF).

**Methods:**

A total of 58 eyes from 32 patients were included, with SS-OCTA examinations conducted preoperatively and 1 day postoperatively. The evaluated parameters encompassed vessel density (VD) of the superficial vascular complex (SVC), VD of the deep vascular complex (DVC), choroidal vascular index (CVI), choroidal thickness (CT), thickness of the superficial retinal layer (SRL), and thickness of the deeper retinal layer (DRL).

**Results:**

Results indicated no significant changes in the VD of the SVC and DVC, nor in the thickness of the superficial and deeper retinal layers. Conversely, CT demonstrated a significant increase (P < 0.05) and the CVI exhibited a notable rise exclusively within the 1-mm diameter region centered on the fovea (P < 0.05).

**Conclusion:**

These findings underscore the remarkable hemodynamic stability of retinal microcirculation in the early postoperative phase. The observed increase in CT and localized elevation in CVI may signify compensatory mechanisms involving enhanced postoperative choroidal perfusion and redistribution of cardiac output, thereby reinforcing the choroid’s superior sensitivity as a biomarker for detecting subtle hemodynamic shifts in AF-related ocular pathology.

## Introduction

Atrial fibrillation (AF) is a common cardiac arrhythmia, with significant risk factors including hypertension, coronary artery disease, heart failure, diabetes, obesity, sleep apnea, and genetic predispositions (Boriani and Pettorelli, 2016; Sagris et al., 2021). With population aging and changes in lifestyle, the incidence and prevalence of AF have been steadily increasing worldwide, significantly impacting public health. It is estimated that by 2050, in China, 5.2 million men and 3.1 million women aged over 60 will be affected by AF, and this number is expected to increase to 7.5 million in America (Naccarelli et al., 2009; Krijthe et al., 2013; Lippi et al., 2021; Zhang et al., 2021; Brundel et al., 2022).

AF is characterized by rapid and irregular contractions of the atria, leading to the loss of practical atrial pumping function (Callizo et al., 2017; Bakhoum et al., 2023; 2024). This condition is often accompanied by fluctuations in cardiac output and systemic hemodynamic alterations, which increase the risk of significant events such as stroke and heart failure and potentially affect abnormal perfusion and microcirculatory changes in multiple organs throughout the body (Brachmann et al., 2021; Lagha et al., 2024; Rossi et al., 2024). Symptoms typically include palpitations, fatigue, dyspnea, and dizziness, although some patients may remain asymptomatic (silent AF). Diagnosis relies on electrocardiography (ECG) or 24-h Holter monitoring (Reddy et al., 2022). Current treatments primarily include anticoagulant therapy to prevent stroke, rate control (such as β-blockers or calcium channel blockers), and potential electrical cardioversion or catheter ablation procedures (Joglar et al., 2024). Radiofrequency catheter ablation (RFCA), as an effective rhythm control strategy, has become an important treatment method for improving heart rhythm and prognosis, as well as reducing complications in patients with AF (Parameswaran et al., 2021; Boersma, 2022). Studies have shown that successful radiofrequency ablation can stabilize sinus rhythm, improving hemodynamic status and enhancing microcirculatory perfusion in organs (Kalla et al., 2017; Parameswaran et al., 2021; Lima-Conceição et al., 2023; Osorio et al., 2023).

With the widespread adoption of advanced ophthalmic imaging technologies such as optical coherence tomography angiography (OCTA), it is now possible to rapidly and non-invasively visualize retinal and choroidal structures and quantitatively assess changes in the retinal microvascular bed (Waheed et al., 2023; Tan et al., 2024; Weerts et al., 2024; Wan et al., 2025). Fundus microcirculation uniquely reflects the state of systemic blood vessels, serving as a “window” for observing the condition of small blood vessels throughout the body (Meng et al., 2023; Waheed et al., 2023; Wang et al., 2023; Weerts et al., 2024). Subtle alterations in fundus blood flow and vascular structure often indicate systemic vascular endothelial dysfunction, microvascular remodeling, and insufficient blood perfusion at an early stage (Callizo et al., 2017; Bakhoum et al., 2023; Weerts et al., 2024). However, in patients with AF, the relationship between fundus flow parameters and hemodynamic changes has not been thoroughly investigated.

Given the unique accessibility of the retinal and choroidal vasculature as a window into systemic microcirculation, the research aims to provide a novel pathway for the early identification and intervention of potential microvascular injuries and the screening and prognostic evaluation of high-risk patients before and after AF rhythm control. Additionally, it seeks to assess the impact of radiofrequency ablation on ophthalmic microcirculatory status and to incorporate ophthalmic assessments into future AF management strategies. The study endeavors to enhance comprehensive patient care and improve overall health outcomes by integrating ophthalmic evaluations in managing AF.

## Materials and methods

### Inclusion criteria

This study was conducted following tenets of the Declaration of Helsinki and was approved by the Ethical Review Committee of Huashan Hospital of Fudan University. Informed consent was obtained for all participants before recruitment. The informed consent was signed by the participants themselves.

Including patients diagnosed with AF who are scheduled to undergo radiofrequency ablation procedures between March 2024 and July 2024 after the use of anti-arrhythmic drugs has become ineffective. All diagnoses were made by an experienced specialist physician (W.J.) after comprehensive examinations of all patients. Patients would be excluded if they had: 1) Systemic hemodynamic instability, impaired mobility and inability to cooperate with the examiner; 2) a history of retinal pathology, such as epiretinal membrane, diabetic retinopathy, or retinoschisis; 3) a history of intraocular surgery; 4) Ocular media opacity or motion artifacts preventing high-quality imaging or stratified artifacts that cannot be manually corrected; 5) ocular hypertension with intraocular pressure >21 mmHg; 6) axial length >26 mm; 7) OCTA images with signal strength less than 7/10. Participant-reported ocular conditions (e.g., history of ocular surgery, macular degeneration, etc.) were confirmed during the ophthalmologic examination.

### Data collection

All subjects’ demographic and clinical characteristics were recorded, including age, sex, history of smoking and alcohol consumption, past medical history (such as diabetes and hypertension), and echocardiography-related data. All patients underwent ophthalmic examinations within 2 hours of admission, which included visual acuity, intraocular pressure (IOP), axial length (AL), and OCTA. Prior to the examinations, subjects rested quietly for 15 min. OCTA assessments were conducted between 10:00 a.m. and 4:00 p.m. to minimize the impact of diurnal variations in choroidal structures. An experienced ophthalmologist evaluated the retinal images to diagnose the presence or absence of retinal lesions. (Y.L.).

### Optical coherence tomography angiography

All patients underwent SS-OCTA (BM400K, TowardPi Medical Technology Co. Ltd. Beijing, China) examinations preoperatively and 24 h postoperatively. Utilizing a scanning source with a wavelength of 1,060 nm and a scanning rate of 400,000 A-scans per second, three-dimensional OCTA images centered on the fovea were acquired, covering a vertical area of 12 mm × 12 mm with a scanning depth of 3 mm. In our study, the following OCTA parameters were analyzed: vessel density (VD) of the superficial vascular complex (SVC), VD of the deep vascular complex (DVC), choroidal thickness (CT), choroidal vascular index (CVI), thickness of superficial retinal layer (SRL) and thickness of deeper retinal layer (DRL).

According to the Early Treatment Diabetic Retinopathy Study (ETDRS) guidelines, the macula is divided into three subregions. The foveal zone is defined by a circle with a diameter of 1 mm centered on the macular center. The parafoveal zone is the annular region between circles with diameters of 1 mm and 3 mm. The perifoveal zone is the annular region between circles with diameters of 3 mm and 6 mm.

**FIGURE 1:**
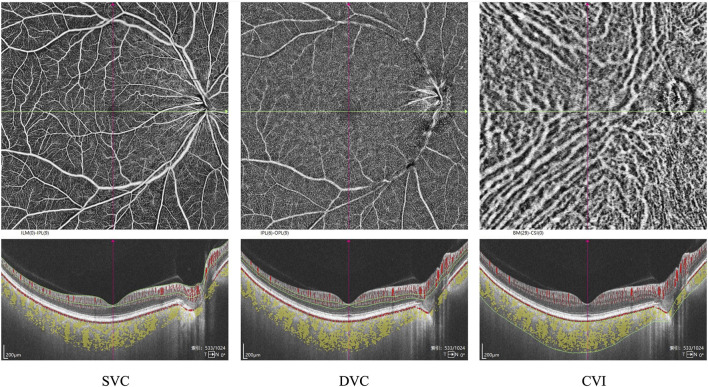
Segmentation of the macular subregions, structure, and microvasculature. SVC: superficial vascular complex, DVC: deep vascular complex, CVI: choroidal vascular index.

**FIGURE 2:**
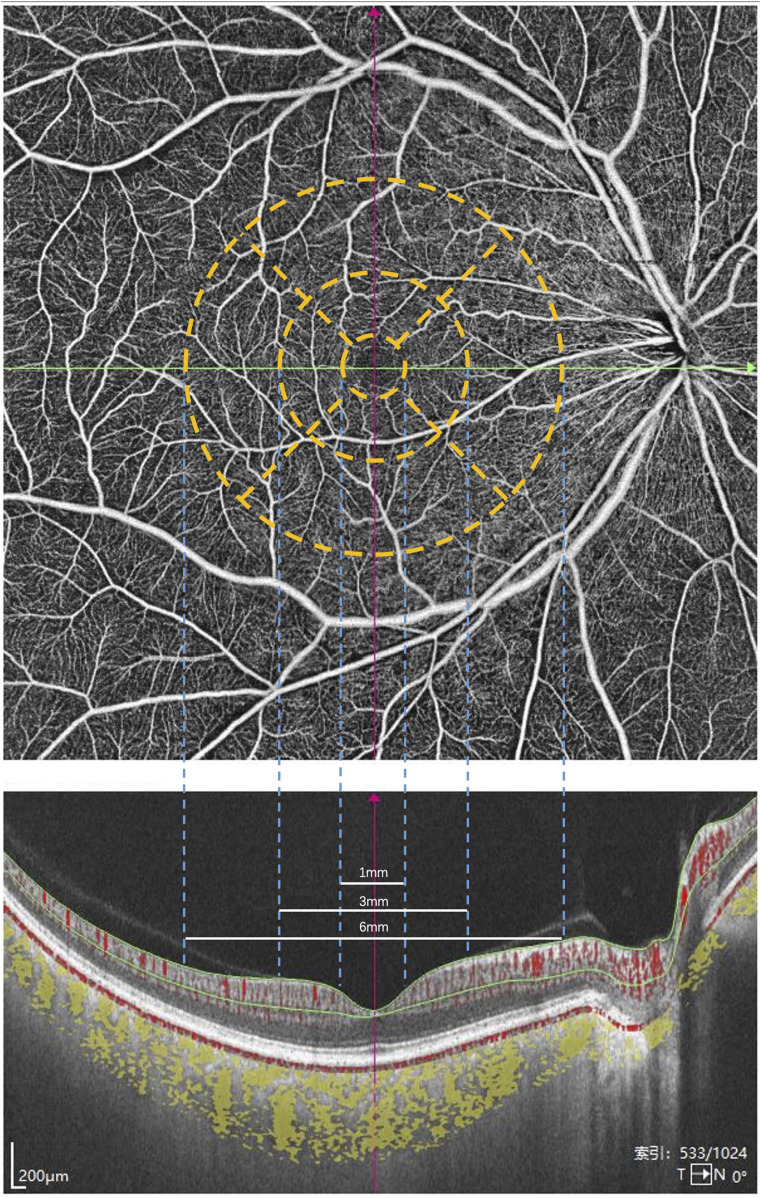
The macula is divided into three subregions according to the ETDRS guidelines. The foveal zone is defined by a circle with a diameter of 1 mm centered on the macular center. The parafoveal zone is the annular region between circles with diameters of 1 mm and 3 mm. The perifoveal zone is the annular region between circles with diameters of 3 mm and 6 mm.

CT was defined as the distance between Bruch’s membrane (BM) and the choroid-scleral interface. Segmentation of BM and the choroid-scleral interface was automatically detected using built-in software. The boundaries of the choriocapillaris layer were delineated using built-in algorithms, extending from BM to 29 µm below BM. If necessary, manual adjustments were performed to correct segmentation errors. Volume projection artifact removal methods were employed to minimize artifacts. VD was defined as the percentage of the area occupied by vascular projections in a given retinal projection image. The SVC refers to the microvasculature from the internal limiting membrane (ILM) to the junction of the inner plexiform layer (IPL) and the inner nuclear layer (INL). In contrast, the DVC refers to the microvasculature from the IPL/INL junction to 25 µm below the outer plexiform layer (OPL). The SRL refers to the distance between the ILM to IPL. The DRL refers to the distance between IPL to the BM (Figure 1). The three-dimensional CVI was calculated as the ratio of choroidal vascular volume to total choroidal volume, primarily reflecting the vascular density of the more extensive vessel layers. All the aforementioned indices were automatically measured and calculated using built-in software, and the platform’s integrated ETDRS rings were applied to compare different regions of the retina (Figure 2). Subsequently, the SS-OCTA platform graded the images on a scale from 1 to 10, and only images with a grade of ≥7 were included in this study. All images were inspected and calibrated by an experienced clinician (Y.H.R.). In cases where automatic segmentation proved inaccurate, choroidal segmentation was meticulously inspected and manually corrected, addressing primarily the misalignment of choroidal boundaries and the decentering of the ETDRS grid.

### Statistical analysis

All analyses were conducted using SPSS version 27.0 for Mac (SPSS Inc. Chicago, IL, United States). The Shapiro-Wilk test was employed to assess the normality of all variables. For patients with bilateral measurements, mixed-effects models were employed to control within-subject correlations. All statistical analyses were two-tailed, and a p-value of less than 0.05 was considered statistically significant.

## Results

In Table 1, a total of 32 patients (58 eyes) were included in this study, comprising 34 male eyes and 24 female eyes. The ages of the participants ranged from 22 to 79 years, with a mean age of 66.74 ± 9.2 years. A history of smoking was present in 39 eyes, hypertension in 44 eyes, diabetes in 50 eyes, and hyperlipidemia in 43 eyes. The average axial length was 23.55 ± 0.961 mm, the mean left ventricular ejection fraction was 61.207% ± 6.712%, the average blood glucose level was 5.55 ± 1.39 mmol/L in the case of regular medication, the mean B-type natriuretic peptide (BNP) was 220.2 ± 334.06 pg/mL, and the D-dimer level averaged 0.39 ± 0.33 μg/mL.

**TABLE 1:** Demographic and clinical characteristics.

Parameter	Object (n = 58)
Demographics	
Age(y),mean ± SD (range)	66.74 ± 9.2 (22–79)
Sex (M/F),n (%)	34/24
Smoking, n (%)	39 (67)
Hypertension,n (%)	44 (76)
Diabetes,n (%)	50 (86)
Hyperlipemia,n (%)	43 (74)
Ancillary tests, mean ± SD (range)	
Axis length	23.55 ± 0.961 (21–26)
LVEF	61.207 ± 6.712 (53–87)
Creatinine	74.06 ± 31.69 (43–215)
Blood glucose	5.55 ± 1.39 (4.0–10.3)
HDL	1.43 ± 0.64 (0.81–4.16)
LDL	2.91 ± 0.93 (0.45–4.79)
BNP	220.2 ± 334.06 (6.0–1676.0)
C-reactive protein	2.78 ± 4.84 (0.80–23.85)
D-dimer	0.39 ± 0.33 (0.07–1.39)

SD, standard deviation; LVEF, left ventricular ejection fraction.

**FIGURE 3:**
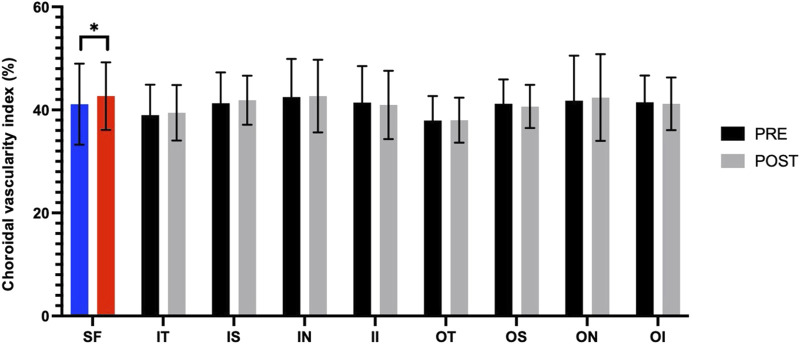
CVI in nine macular regions of the ETDRS grid before and after the procedure.

**FIGURE 4:**
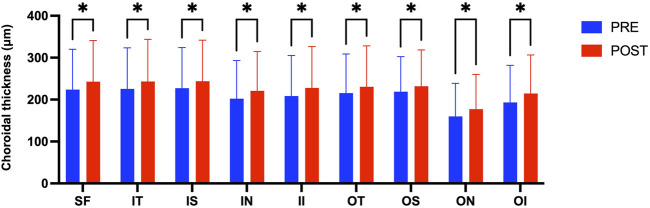
CT in nine macular regions of the ETDRS grid before and after the procedure.

**FIGURE 5:**
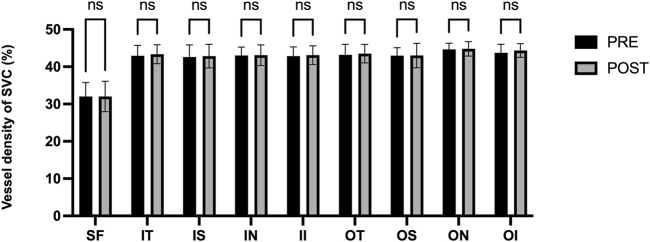
Blood flow density in the superficial retinal layers before and after the procedure.

**FIGURE 6:**
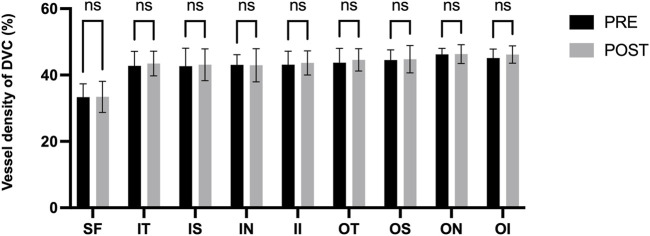
Blood flow density in the deep retinal layers before and after the procedure.

**FIGURE 7:**
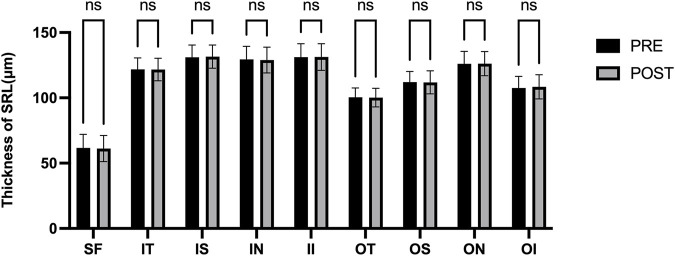
Thickness of SRL in nine macular regions of the ETDRS grid before and after the procedure.

**FIGURE 8:**
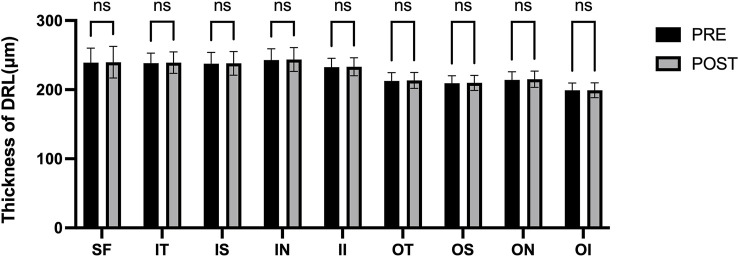
Thickness of DRL in nine macular regions of the ETDRS grid before and after the procedure.

Figure 3 presents the measurements of the CVI in all macular regions before and after the procedure. The post-operative group exhibited a higher CVI in the subfoveal choroid compared to the pre-operative group, with the difference being statistically significant (P < 0.05). No significant differences were observed in the other regions (P > 0.05). Figure 4 displays the measurements of CT before and after the procedure. All subregions showed a significant increase in CT post-operatively compared to pre-operatively (P < 0.05), indicating statistically significant differences. Figures 5, 6 show the blood flow density in the superficial and deep retinal layers before and after the procedure. Only the inferior outer region demonstrated a statistically significant difference, while the other subregions showed no significant differences (P > 0.05). Figures 7, 8 displays the measurements of thickness of SRL and DRL before and after the procedure which both showed no significant differences (P > 0.05).

## Discussion

AF is a disease that can alter systemic hemodynamics. Its associated hemodynamic disturbances can adversely affect microvascular circulation in various organs (Kawasaki et al., 2012; Christiansen et al., 2013; Gopinath et al., 2019; Daxer et al., 2024; Weerts et al., 2024). OCTA is related to the signal intensity and can non-invasively and quickly visualize the fundus blood circulation, which makes OCTA an ideal method for assessing fundus blood flow in patients with AF.

In this study, we used swept-source OCTA to evaluate the potential hemodynamic changes of the retina and choroid in patients who underwent radiofrequency catheter ablation. We observed almost no significant changes in the hemodynamic parameters of the retina (VD of SVC and DVC, thickness of SRL and DRL) or choroid (CVI) in examined macular subregions. However, CT increased significantly in all the subregions following surgery.

Post-operative assessments demonstrated a statistically significant increase in subfoveal CVI and CT, indicating that the choroidal hemodynamics is comprehensively improved after RFCA. This change may be related to the stabilization of cardiac output and the optimization of systemic hemodynamics after RFCA restores sinus rhythm. Philipp S Lange et al. found the flow density in the optic nerve head (ONH) radial peripapillary capillary network improved significantly in the study group following pulmonary vein isolation (PVI) in patients with AF. Rhythm control using PVI significantly improved ocular perfusion as measured using OCT-A (Lange et al., 2021). The increase in CT potentially reflects the congestion of choroidal perfusion after the operation. In a study conducted by Rakusiewicz et al., it was observed that pediatric patients suffering from congestive heart failure (HF) caused by dilated cardiomyopathy exhibited reduced CT across all measured regions. The researchers concluded that chronic HF significantly influences CT, suggesting that this metric could serve as a valuable indicator for tracking the clinical progression of cardiovascular disease (Rakusiewicz et al., 2021).

Although the CT in all directions of the macula generally increased, only the CVI under the fovea increased significantly, suggesting that the choroidal vascular response in the foveal area is more sensitive (Linsenmeier and Zhang, 2017; Fernández-Vigo et al., 2020). The choroid is a highly vascularized tissue structure and one of the tissues with the highest blood flow in the body, accounting for approximately 85%–90% of the blood flow from the ophthalmic artery. These arterial branches form the choroidal vascular network, directly supplying oxygen and nutrients to the outer layers of the retina. The fovea, however, is an avascular region, and its nutrition entirely relies on the diffusion from the choroidal capillaries (Wangsa-Wirawan and Linsenmeier, 2003; Mrejen and Spaide, 2013; Ferrara et al., 2016; Borrelli et al., 2018). Any disease that disrupts blood flow to the choroid or retina—such as age-related macular degeneration or diabetic retinopathy—can lead to macular dysfunction. In recent years, numerous studies have uncovered significant associations between choroidal health and a wide range of systemic diseases, highlighting its potential role in systemic pathophysiological processes (Dervisogullari et al., 2015; Aydin et al., 2017; Kim et al., 2021; Rakusiewicz et al., 2021; Robbins et al., 2021). Liu found that CT decreased more rapidly in the macular region relative to the other areas after 50 years old using SS-OCTA (Liu X. et al., 2023). Wei et al. found that SFCT was significantly associated with higher diastolic blood pressure and higher mean blood pressure, and with the presence of arterial hypertension in univariate analysis (Wei et al., 2013). The increase in CVI of subfoveal may reflect an enhancement in the choroidal vessel density or an optimization of blood flow distribution in this area, further supporting the important role of choroidal vessels in providing nutritional support to the avascular zone of the fovea (Gonzales et al., 2025). Rupesh Agrawal et al. found that compared to CT, the CVI exhibits lower covariance and is independent of patient factors such as age, systolic blood pressure, axial length, or intraocular pressure (Agrawal et al., 2016). The review by Motamed Shariati M et al. highlighted a significant correlation between variations in the CVI and a range of systemic diseases that impact hemodynamics (Motamed Shariati et al., 2024).

The retinal thickness both in SRL and DRL did not change significantly after the operation, indicating that the retinal microcirculation maintained remarkable homeostasis after RFCA. This result may be related to the self - regulatory ability of retinal blood vessels, enabling them to maintain a relatively stable blood supply amidst systemic hemodynamic changes (Yuan et al., 2024). Yu et al. found Retinal capillary perfusion is significantly heterogeneous in both space and time. This heterogeneity allows the limited blood supply to be distributed more efficiently to areas with higher metabolic demands (Yu et al., 2019). In addition, there were no significant changes in the VD of the SVC and the DVC, further supporting the stability of the retinal microcirculation in the early postoperative period. This finding suggests that RFCA has minimal direct impact on the retinal structure, and the retinal microcirculation exhibits strong tolerance and regulatory capacity. Liu et al. found that individuals with AF had decreased retinal vascular densities and perfusion in SVC, as well as thinner GCIPL and RNFL thickness compared with age- and sex-matched control participants (Liu J. et al., 2023). Bengi Ece Kurtul et al. found that although foveal retinal thickness and nasal retinal nerve fiber layer thickness are affected in patients undergoing catheter ablation for ventricular arrhythmia, the stable retinal and optic disc vessel densities can be explained by the administration of effective anticoagulants during the procedure (Kurtul et al., 2023). The mechanism by which retinal thickness and blood flow density remain stable after surgery needs to be further explored.

At present, OCTA is widely used to connect fundus microcirculation and systemic diseases (Kalra et al., 2022; Waheed et al., 2023). Fu’s study revealed a significant association between retinal microvascular parameters and incident CHD. As the lower complexity and density of the retinal vascular network may indicate an increased risk of incident CHD, this may empower its prediction with the quantitative measurements of retinal structure (Fu et al., 2023). Gen-Min Lin et al. observed an association in women, but not men, of wider retinal venular calibers with the incidence of AF by retinal photography and retinal grading. The reasons for a possible interaction are incompletely understood (Lin et al., 2021). Christine Y. Bakhoum et al. demonstrated that retinal ischemic perivascular lesions (RIPLs), which are indicative of ischemia in the middle retina., are significantly associated with AF, independent of underlying ischemic heart disease or cardiovascular risk factors (Bakhoum et al., 2023). Indrė Matulevičiūtė et al. found that a decrease in retinal and CT, as well as decreased vascular density in the central retinal region, may predict coronary artery disease (Matulevičiūtė et al., 2022).

The observed improvement in CVI and CT post-RFCA is consistent with research suggesting that restoring sinus rhythm can ameliorate systemic hemodynamic parameters, thereby enhancing microvascular perfusion. To the best of our knowledge, no previous studies have demonstrated that AF ablation can improve retinal blood flow. Our study is the first to provide evidence supporting the hypothesis that RCFA not only stabilizes cardiac rhythm but also positively influences ocular blood flow parameters, thereby offering a more comprehensive understanding of the systemic benefits of rhythm control strategies in AF management. These findings highlight the importance of incorporating ophthalmic evaluations in the pre- and post-operative assessment of AF patients undergoing RFCA. Monitoring retinal and choroidal microcirculation could serve as a non-invasive indicator of systemic vascular health and the effectiveness of rhythm control strategies. More and more AI tools are being used to study eye diseases associated with systemic diseases in order to guide more comprehensive treatments (Guang-Hua Zhang and Guang-Hua Zhang, 2024; Yi et al., 2024). Early detection of microvascular changes may facilitate timely interventions to prevent ocular and systemic complications in high-risk AF patients.

This study has several limitations. The relatively small sample size may limit the generalizability of our findings, particularly in diverse patient populations with varying degrees of AF severity and comorbidities. Future studies with larger cohorts are needed to validate these results. In addition, continuous cardiac output monitoring and complete ECG data were not collected before and after surgery in this study, and it is hoped that the data collection will be further improved in future related studies.

Future studies with larger cohorts and longitudinal follow-up are necessary to validate these results and explore the long-term effects of RCFA on ocular microcirculation using OCTA (Javed et al., 2023). Future research should aim to include a larger and more diverse patient population to enhance the robustness of the findings. Longitudinal studies could provide insights into the temporal relationship between RCFA and ocular microvascular changes.

## Data Availability

The original contributions presented in the study are included in the article/supplementary material, further inquiries can be directed to the corresponding author.
